# Effects of a 9-month resistance training intervention on quality of life, sense of coherence, and depressive symptoms in older adults: randomized controlled trial

**DOI:** 10.1007/s11136-017-1733-z

**Published:** 2017-11-09

**Authors:** Tiia Kekäläinen, Katja Kokko, Sarianna Sipilä, Simon Walker

**Affiliations:** 1 Gerontology Research Center and Faculty of Sport and Health Sciences, University of Jyväskylä, PO Box 35, 40014 Jyväskylä, Finland; 2 Neuromuscular Research Center and Faculty of Sport and Health Sciences, University of Jyväskylä, PO Box 35, 40014 Jyväskylä, Finland

**Keywords:** Quality of life, Aging, Exercise, Well-being, Coping, Mood

## Abstract

**Purpose:**

(1) To determine the effects of a 9-month resistance training intervention on quality of life, sense of coherence, and depressive symptoms in older adults, and (2) to compare effects between different training frequencies.

**Methods:**

Men and women aged 65–75 (*N* = 106) were randomized to four groups according to training frequency: training groups RT1 (*n* = 26), RT2 (*n* = 27), and RT3 (*n* = 28) and non-training control group (*n* = 25). All training groups attended supervised resistance training twice a week for 3 months. For the following 6 months, they continued training with different frequencies (1, 2 or 3 times per week). Psychological functioning was measured by quality of life (WHOQOL-Bref), sense of coherence (Antonovsky’s SOC-13), and depressive symptoms (Beck’s Depression Inventory II). Measurements were conducted at baseline and 3 and 9 months after baseline. The effects of the intervention were analyzed using generalized estimating equations (GEE).

**Results:**

After 3 months, there was an intervention effect on environmental quality of life (group × time *p* = .048). Between 3 and 9 months, environmental quality of life decreased among RT1 compared to RT2 and RT3 (group × time *p* = .025). Between baseline and 9 months, environmental quality of life increased in RT2 compared to all other groups (group × time *p* = .011). Sense of coherence increased in RT2 compared to the control group and RT3 (group × time *p* = .032).

**Conclusion:**

Resistance training is beneficial for environmental quality of life and sense of coherence. Attending resistance training twice a week seems to be the most advantageous for these aspects of psychological functioning.

## Introduction

Quality of life (QoL) is a major area of interest in a wide range of fields. The World Health Organization [1] has defined QoL as “individuals’ perception of their position in life in the context of the culture and value systems in which they live and in relation to their goals, expectations, standards and concerns.” QoL is a subjective evaluation, which is related to an individual’s cultural, social, and environmental context, and encompasses four different domains: physical, psychological, social, and environmental [1]. Of these domains, especially the physical domain tends to decrease with age [2].

Sense of coherence (SoC) and depressive symptoms (DS) are both closely related to QoL [3–5]. According to Antonovsky’s salutogenesis theory [6], SoC is a life orientation and reflects an individual’s perception of how meaningful, manageable, and comprehensible their life is. SoC could be seen as a health resource, because it reveals how people perceive life and use their resources to cope with stressors. Both cross-sectional and longitudinal studies [3] have shown SoC to be related to QoL. Unlike SoC, depression is a major mental health problem [7]. Even minor levels of depression are related to poorer QoL in all domains [4, 5] and to weaker SoC [8]. Hence, both SoC and DS are worth considering when promoting older adults’ QoL.

Physical activity is a key factor to maintain health and functional capacity during aging. Both aerobic physical activity (e.g., at least 150 min/week in moderate intensity) and muscle-strengthening activities (at least twice a week) are recommended activities for older adults [9]. Only a minority of older adults meet these recommendations, especially for muscle-strengthening activities [10]. This is alarming, because resistance training (RT) offers major benefits, such as improving strength and power, increasing muscle mass, reducing risk of functional limitations, difficulties in daily tasks and falls, and enables individuals to stay physically active [11, 12]. In addition to physical benefits, earlier literature has shown that exercise is positively associated with psychological functioning, such as QoL and DS, in older adults [13–16]. The underlying reasons why exercise was shown to be beneficial to psychological functioning may have been related to improvements in physical functioning (e.g., muscle strength, cardiovascular functioning), social interaction, or mastery experiences gained during exercise [14, 17], for example.

The effect of RT on QoL is still unclear; in some intervention studies RT appeared to have a positive effect especially on the physical domain of QoL [18–20], whereas in others no effects were found [21–23]. Positive effects have been found both after shorter (e.g., 12 weeks) [18, 19] and longer (e.g., 8 months) [20] RT interventions. Only few studies have reported the effects of RT on SoC. Among older adults with hip fracture history, a 12-week strength training intervention (training twice a week) did not influence SoC [24], whereas another 10-month strength/flexibility/balance training intervention study (training three times a week) had a positive within-group effect on SoC [25]. Previous studies are more consistent regarding DS; evidence shows that RT slightly reduces DS in both healthy older adults and those with diagnosed depression [26, 27]. Therefore, it seems that RT may have a positive influence on psychological functioning, but more studies are needed to uncover the effects of RT on specific areas of psychological functioning among older adults.

Another unclear area is the effect of RT frequencies on psychological functioning. One earlier study compared the effect of RT two or three times a week [28] in older women and another study RT three or four times a week [29] on QoL in middle-aged women. In these two studies, both intervention groups improved their QoL without significant between-group differences. However, the durations of the interventions were 12 [28] and 8 [29] weeks, so it is not clear whether training frequencies have different effects after long-term RT. In addition, training frequency has usually been two or three times a week and it is unclear whether RT only once a week could have an effect on psychological functioning.

The purpose of this study was to (1) investigate the effects of RT intervention on psychological functioning, assessed here by QoL, SoC, and DS, in older adults after 3 and 9 months of training and (2) compare the effect of different training frequencies (one, two, or three times a week) on these areas of psychological functioning. The present study was based on a secondary analysis of a randomized controlled trial investigating the minimum training frequency to improve neuromuscular performance and health among older adults. Physical performance results from the same trial show that RT increased maximum strength [30] and cardiorespiratory fitness after 3 months of training [31], and a higher training frequency provided greater benefit for maximum dynamic strength but not for functional capacity over 9 months [32]. Based on those results and previous studies on psychological functioning, we hypothesized that RT is beneficial for psychological functioning already after 3-month training and there are no differences between frequencies.

## Methods

### Study design and participants

**Fig. 1:**
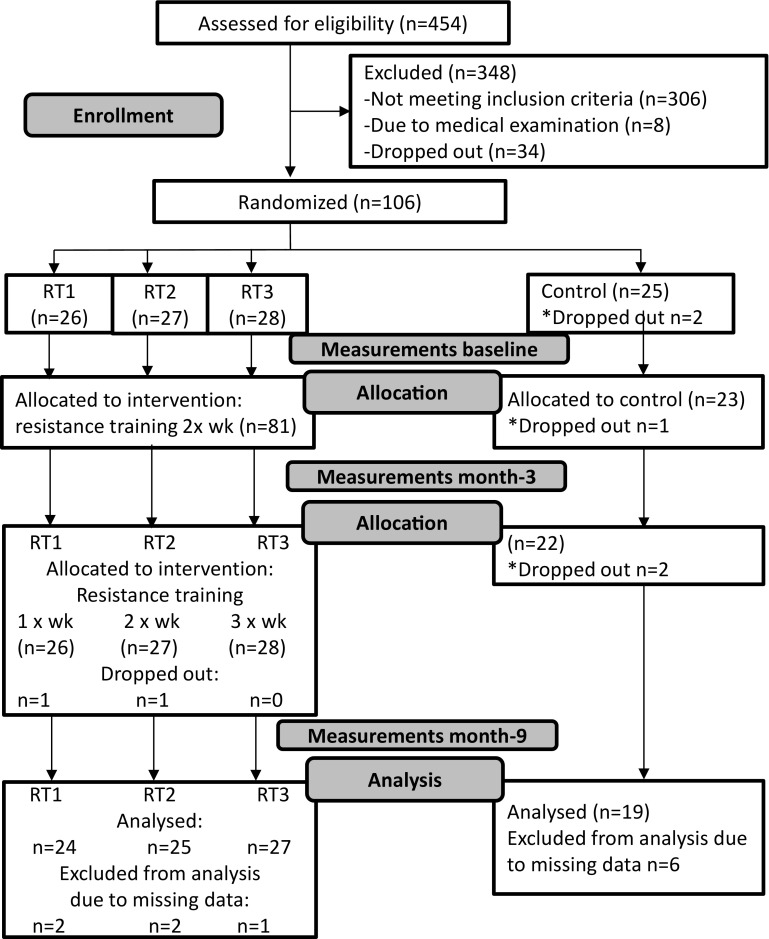
Flow diagram of the study

The present study was based on a secondary analysis of a parallel-group randomized controlled trial, “Minimum resistance training frequency: effect on motivation and adherence to train, overall health status and neuromuscular performance” (NCT02413112). The trial is described in further detail in previous studies [30–32]. The flowchart of the study is shown in Fig. 1. Pre-trial statistical power analysis was performed for the primary outcomes, maximum strength, and functional capacity, based on the effect sizes reported in a meta-analysis by Liu and Latham [33]. With a 75:25 intervention-to-control ratio, a sample size of 44 (intervention) and 15 (control) for strength and 66 and 22 for functional capacity was necessary to reach 80% probability that treatment differences could be observed with a 5% level of significance. Two thousand invitation letters were sent to a random sample of community-dwelling 65–75-year-old older adults living in the Jyväskylä area. In total, 454 (23%) responded by filling in an online registration form. The exclusion criteria were (1) regular aerobic exercise (over 3 h/week), (2) RT experience, (3) BMI > 37, (4) previous testosterone-altering treatment, (5) serious cardiovascular disease that may lead to complications during exercise, (6) use of pharmaceuticals that affect the neuromuscular or endocrine systems, (7) use of walking aids, and (8) smoking. Potential participants (*n* = 148 of 454) were invited to an information session. One hundred and sixteen persons provided written informed consent and attended a doctor’s examination to assess their health and ability to perform RT; eight persons were excluded due to medical reasons. After two drop-outs, 106 participants were randomized by a random number generator (online) in a block of 100 subjects, so that 25 subjects were selected into each group. Each number in sequence was allocated to each group in descending order. The remaining six subjects were randomized within the training groups. As it was assumed that training three times a week likely increased the chance of drop-out and/or non-compliance, three subjects were randomized to group 3, two to group 2, and one to group 1. The study groups were RT once a week (RT1, *n* = 26), twice a week (RT2, *n* = 27), three times a week (RT3, *n* = 28), and non-training control group (CG, *n* = 25). After randomization, two participants from CG dropped out because they were dissatisfied with the results of randomization. Hence, 104 participants started the study. CG was instructed not to change their lifestyle during the intervention, and after post-intervention measurements, they had an opportunity to participate in supervised RT twice a week for 6 months. Ethical approval was obtained from the University of Jyväskylä Ethical Committee.

### Intervention

All training sessions were performed at the gym in the University of Jyväskylä, and were supervised by experienced personnel. Each training session lasted for 1 h, and consisted of a 10-min warm-up and 8–9 exercises for different muscle groups. *Months 1–3*: All three training groups trained twice a week, to become familiar with RT methods, and to build capacity for subsequent high-load training. The focus was on local muscular endurance using low loads. *Months 4–9*: The training groups split into different training frequencies in which they were randomized: For RT1, this represented a reduced and for RT3 an increased training frequency. All training groups followed identical two-session training programs: completing one cycle took 2 weeks for RT1, 1 week for RT2, and RT3 completed three cycles in 2 weeks. Training during months 4–9 was progressively periodized RT, focused on muscle hypertrophy and maximum strength.

### Measurements

#### Quality of life

QoL was assessed using the WHOQOL-BREF questionnaire, which is a shortened version of the WHOQOL-100 [1]. The WHOQOL-BREF is a valid and reliable method to measure QoL in older adults [2, 34]. WHOQOL-BREF includes 26 questions and covers all four domains of QoL. The participants scored the items on a scale from 1 to 5, and the raw domain scores were transformed to a scale of 4–24. Cronbach’s alphas for physical domain were .70, .68, and .70, for psychological domain .77, .76, and .77, for social domain .67, .78, and .77, and for environmental domain .65, .75, and .79 at baseline, 3, and 9 months, respectively.

#### Sense of coherence

SoC was measured by Antonovsky’s 13-item scale, derived from the original 29-item scale [6, 35]. SoC scale is a widely used reliable and valid measurement [35]. The answers were given on a scale from 1 to 7, and aggregated. Cronbach’s alphas were .77, .80, and .82 at baseline, 3, and 9 months, respectively.

#### Depressive symptoms

DS were assessed with the Beck Depression Inventory II (BDI-II), which is a revised version of the original BDI [36]. The BDI-II has adequate reliability and validity in all age groups [37]. The inventory consists of 21 statements, which are scored from not present (0) to severe (3), and the overall scoring range is 0–63. Cronbach’s alphas were .72 at baseline, .77 at month-3, and .77 at month-9.

#### Aerobic exercise

The mean amount (minutes/week) of aerobic exercise at baseline was obtained by a self-reported questionnaire. Physical activity diaries tracked daily leisure-time aerobic exercise throughout the study. The average weekly aerobic exercise in months 1–3 and 4–9 was calculated.

#### Strength and functional capacity

The protocols to measure strength and functional capacity are described previously [30–32]. A bilateral leg press one-repetition maximum (1-RM) was used to assess maximum strength. Functional capacity was assessed by time to complete 7.5 m forward and backward walk, timed-up-and-go (TUG), and loaded 10-stair climb tests.

### Statistics

Analyses were performed using IBM SPSS Statistics 24.0 (IBM Corp., released 2016, IBM SPSS Statistics for Windows, Armonk, NY). Results presented here were analyzed by intention-to-treat principle with the exception of those two participants who dropped out from CG before baseline measurements right after the randomization.

The effect of the intervention on QoL, SoC, and DS was analyzed by generalized estimating equation (GEE) models with an unstructured working correlation matrix. The advantage of the GEE method is that when pre- or post-intervention measurement is missing, it utilizes the information also from incomplete pair of observations [38]. Three sets of GEE models were performed. First, the differences between the training group and CG from baseline to month-3 were tested. Second, the differences between CG, RT1, RT2, and RT3 from month-3 to month-9 were tested, and third, the differences between these four groups from baseline to month-9 were tested.

The amount of leisure-time aerobic exercise (min/week) was not stable in all groups during the intervention. CG had significantly higher amount of aerobic exercise in months 1–3 (mean 193 min/week) and 4–9 (172 min/week) compared to baseline (110 min/week) (paired sample t-test *p* = .028 and *p* = .039, respectively), and RT3 in months 1–3 (145 min/week) compared to baseline (85 min/week) (*p* = .007). Therefore, GEE analyses were adjusted by average aerobic exercise (min/week). Leisure-time physical activity diaries from months 1–3 were missing from 19 participants, of which 11 were from CG. The missing data for CG participants were imputed using linear regression imputation, with baseline and 4–9 months of aerobic exercise as predictors, for those (*n* = 7) who had both values. After the imputation, the final sample sizes for GEE analyses were 19/23 in CG and 73/81 in the training group for baseline to month-3 analyses (9% missing) and 19/23 in CG, 24/26 in the RT1, 25/27 in the RT2, and 27/28 in the RT3 for month-3 to month-9 and baseline to month-9 analyses (9% missing).

Changes in outcome variables were calculated by subtracting the previous value from the intervention completion value. The standardized effect sizes for differences between groups were calculated with Cohen’s *d* formula [39]. Within-group differences were analyzed by paired sample *t* tests.

## Results

There were one drop-out from CG between baseline and month-3, and four drop-outs (two from CG, one from RT1, and one from RT2) between month-3 and month-9 (Fig. 1). There were no significant differences (*p* > .05) between the groups in participants’ characteristics (Table 1) and QoL, SoC, and DS (Table 2) at baseline.

**Table 1:** Participants’ characteristics at baseline

	CG (*n* = 23)	RT1 (*n* = 26)	RT2 (*n* = 27)	RT3 (*n* = 28)	*p* ^a^
Gender: female (%)	47.8	53.8	59.6	57.1	.864
Age (year)	68.3 (2.3)	68.9 (2.7)	67.7 (2.8)	69.0 (3.3)	.282
Education (%)					.091
Basic comprehensive school	30.4	32.0	15.4	53.6	
Upper secondary education	21.7	32.0	42.3	25.0	
Tertiary education	47.8	36.0	42.3	21.4	
Marital status (%)					.945
Married/cohabitation	73.9	76.0	80.8	78.6	
Single/divorced/widowed	26.1	24.0	19.2	21.4	
Weight (kg)	74.5 (11.6)	76.5 (14.5)	80.6 (14.1)	81.5 (14.7)	.235
Height (cm)	167.5 (8.7)	166.8 (8.7)	167.9 (7.3)	167.4 (9.3)	.976
BMI (kg/m^2^)	26.4 (2.6)	27.3 (3.3)	28.6 (4.4)	29.0 (4.1)	.070
Aerobic training min/week	110.0 (62.7)	113.2 (63.6)	110.8 (56.1)	84.6 (58.3)	.143

Mean and standard deviations or frequencies presented

*CG* = control group, *RT1-3* = resistance training one, two or three times-a-week groups

^a^Differences between groups tested by ANOVA for continuous variables and Chi-Square test for categorical variables

**Table 2:** Means and standard deviations (SD) of quality of life (QoL), sense of coherence (SoC), and depressive symptoms (DS) at baseline, month-3, and month-9, and effect sizes for mean changes between the groups

	Baseline mean (SD)	Month-3 mean (SD)	Month-9 mean (SD)	∆Effect size (95% CI) 0–3	∆Effect size (95% CI) 3–9	∆Effect size (95% CI) 0–9
Physical QoL						
CG (*n* = 23, 21, 20)^a^	16.6 (2.1)	16.7 (1.6)	16.7 (1.8)	Ref.	Ref.	Ref.
RT1, RT2 and RT3 (*n* = 78, 79)	16.8 (1.7)	17.2 (1.7)^b^		.44 (− .05, .92)		
RT1 (*n* = 25, 25, 25)	17.2 (1.7)	17.5 (1.7)	17.6 (1.6)		− .09 (− .69, .51)	.31 (− .29, .90)
RT2 (*n* = 26, 27, 26)	16.7 (1.7)	17.1 (1.4)	17.1 (1.7)		− .06 (− .65, .54)	.26 (− .33, .85)
RT3 (*n* = 28, 26, 28)	16.5 (1.8)	16.8 (2.1)	16.6 (2.3)		− .22 (− .81, .38)	.12 (− .46, .69)
Psychological QoL						
CG	15.4 (1.9)	16.1 (1.6)	16.1 (1.5)	Ref.	Ref.	Ref.
RT1, RT2 and RT3	16.1 (2.0)	16.6 (1.9)^b^		.09 (− .40, .57)		
RT1	16.4 (1.9)	16.8 (1.7)	17.1 (1.8)^d^		.08 (− .53, .68)	.30 (− .31, .89)
RT2	16.0 (1.7)	16.5 (1.7)	16.7 (1.8)^d^		.03 (− .56, .62)	.35 (− .25, .94)
RT3	15.9 (2.4)	16.6 (2.3)	16.3 (2.3)		− .18 (− .77, .41)	.17 (− .40, .74)
Social QoL						
CG	14.3 (2.4)	14.6 (2.9)	14.6 (3.0)	Ref.	Ref.	Ref.
RT1, RT2, and RT3	15.2 (2.5)	15.0 (2.9)		.01 (− .47, .49)		
RT1	14.8 (2.7)	14.5 (2.9)	14.6 (2.5)		.05 (− .55, .65)	.09 (− .51, .68)
RT2	15.8 (1.9)	15.1 (2.8)	14.8 (2.4)^d^		− .03 (− .62, .56)	.24 (− .83, .35)
RT3	15.0 (2.8)	15.4 (3.0)	14.1 (2.9)^c^		− .35 (− .94, .25)	− .17 (− .74, .41)
Environmental QoL						
CG	16.6 (1.7)	16.5 (1.7)	16.4 (2.0)	Ref.	Ref.	Ref.
RT1, RT2, and RT3	16.8 (1.7)	17.0 (2.0)		.38 (− .11, .86)		
RT1	17.2 (1.7)	17.7 (1.5)	17.2 (1.6)		− .43 (− 1.03, .19)^e^	.23 (− .37, .82)
RT2	16.8 (1.2)	16.9 (1.7)	17.6 (1.4)^d^		.40 (− .20, .99)	.85 (.23, 1.45)^f^
RT3	16.4 (2.0)	16.5 (2.4)	16.5 (2.3)		.14 (− .45, .73)	.39 (− .19, .97)
SoC						
CG	71.3 (6.5)	72.0 (7.0)	72.5 (7.8)	Ref.	Ref.	Ref.
RT1, RT2, and RT3	72.8 (1.5)	75.5 (9.8)^b^		.30 (− .18, .79)		
RT1	75.8 (10.1)	77.9 (9.6)	79.7 (10.0)^d^		− .06 (− .67, .54)	.48 (− .13, 1.07)
RT2	7.2 (11.2)	74.3 (8.9)	77.0 (7.0)^c, d^		.22 (− .37, .81)	.85 (.22, 1.44)^g^
RT3	72.6 (9.9)	74.4 (10.8)	74.8 (10.3)		− .11 (− .70, .48)	.28 (− .30, .85)
DS						
CG	5.0 (2.9)	4.1 (3.2)^b^	3.6 (3.6)^d^	Ref.	Ref.	Ref.
RT1, RT2, and RT3	4.5 (3.7)	3.3 (3.4)^b^		− .12 (− .60, .36)		
RT1	3.7 (3.7)	3.5 (4.2)	3.4 (3.5)		.19 (− .41 to .79)	.36 (− .24, .96)
RT2	4.7 (3.6)	3.5 (2.6)	3.0 (2.9)^d^		.02 (− .57, .61)	.08 (− .66, .51)
RT3	5.0 (3.8)	2.9 (3.3)	3.1 (3.2)^d^		.34 (− .26, .93)	− .24 (− .82, .34)

Significant (*p* < .05) difference within group between ^b^Baseline and month-3, ^c^Month-3 and month-9, ^d^Baseline and month-9

*CG* control group, *RT1-3* resistance training one, two or three times a week groups, *Ref*. reference group

^a^Sample sizes for baseline, month-3 and month-9, respectively. All outcomes have the same sample size

^e^Effect size compared to RT2 − 0.78 (− 1.34 to − 0.19) and to RT3 − 0.54 (− 1.09 to 0.03)

^f^Effect size compared to RT1 0.60 (0.01–1.16) and to RT3: 0.59 (0.03–1.13)

^g^Effect size compared to RT3: 0.65 (0.09–1.20)

**Fig. 2:**
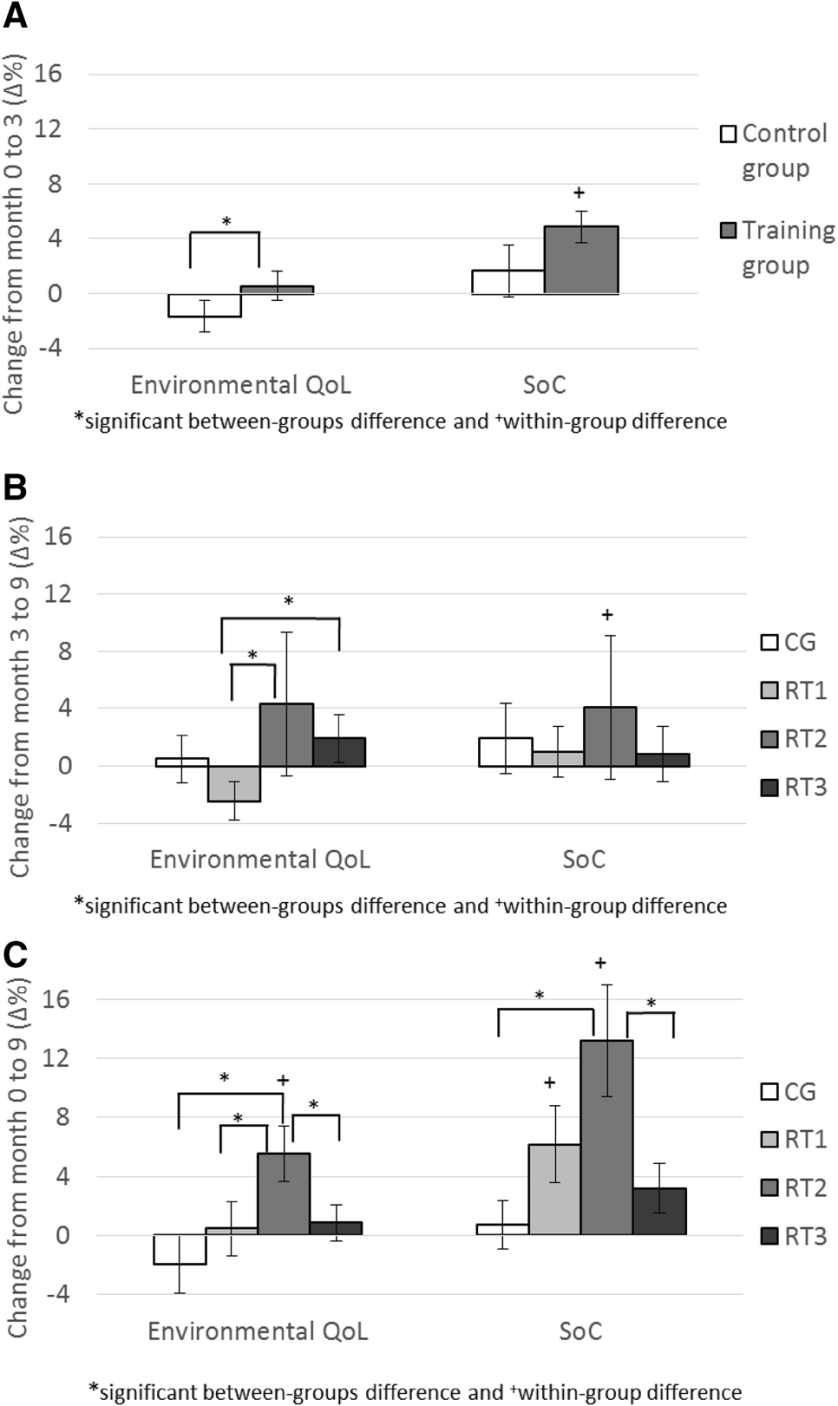
Study key findings: changes in environmental quality of life (QoL) and sense of coherence (SoC) (mean and SE). *CG* control group, *RT1-3* resistance training one-, two-, or three-times-a-week group

Results for QoL, SoC, and DS are presented in Tables 2 and 3. After the 3-month intervention, there were within-group improvements in physical and psychological QoL and SoC in the training group and in DS in both training group and CG. The only significant group × time difference was found in environmental QoL (Table 3; Fig. 2a). From month-3 to month-9, different frequencies were used by the training groups. Social QoL decreased in RT3 and SoC increased in RT2. The only significant change between the groups occurred in the environmental QoL (Table 3; Fig. 2b). Environmental QoL decreased in RT1 compared to RT2 and RT3. From baseline to month-9, psychological QoL and SoC improved in RT1 and RT2, social QoL decreased and environmental QoL increased in RT2, and DS decreased in RT2, RT3, and CG. SoC and the environmental QoL showed a significant group × time interaction (Table 3; Fig. 2c). Throughout the 9-month intervention, both SoC and environmental QoL increased in RT2 compared to CG and to RT3, and environmental QoL also compared to RT1. According to effect sizes (Table 2), the changes in SoC and environmental QoL in RT2 were large compared to CG (effect sizes > 0.80) and medium compared to RT1 and RT3 (effect sizes > 0.50). The correlations between changes in QoL, SoC, DS, strength, and functional capacity are shown in Table 4.

**Table 3:** The effect of intervention on quality of life (QoL), sense of coherence (SoC), and depressive symptoms (DS), analyzed by generalized estimated equations (GEE)

	GEE model 0–3^a^			GEE model 3–9^b^			GEE model 0–9^c^		
	Group *p*	Time *p*	Group × time *p*	Group *p*	Time *p*	Group × time *p*	Group *p*	Time *p*	Group × time *p*
Physical QoL	.570	.087	.064	.276	.662	.828	.291	.323	.685
Psychological QoL	.262	< .001	.814	.439	.832	.421	.161	< .001	.521
Social QoL	.543	.276	.722	.886	.080	.493	.520	.004	.740
Environmental QoL	.578	.308	.048	.076	.469	.025^d^	.260	.228	.011^e^
SoC	.241	.002	.110	.057	.178	.550	.222	< .001	.032^f^
DS	.722	< .001	.840	.912	.348	.429	.979	< .001	.201

Models adjusted by mean aerobic physical activity (min/week) from ^a^months 1–3, ^b^months 4–9, and ^c^months 1–9. ^d^RT1 had significant differences compared to RT2 (*p* = .005) and RT3 (*p* = .036). ^e^RT2 had significant differences compared to CG (*p* = .001), RT1 (*p* = .047) , and RT3 (*p* = .043). ^f^RT2 had significant differences compared to CG (*p* = .006) and RT3 (*p* = .017)

**Table 4:** Pearson’s correlations (*r*-values) between changes in quality of life (QoL), depressive symptoms (DS), sense of coherence (SoC), strength and functional capacity: changes between baseline and month-3 above diagonal and between baseline and month-9 below diagonal

Absolute change	1	2	3	4	5	6	7	8^a^
1. Physical QoL	–	.33**	.18	.37***	− .23*	.18	.21*	− .00
2. Psychological QoL	.42***	–	.08	.25*	− .25*	.26**	.08	− .02
3. Social QoL	.23*	.13	–	.12	− .03	− .10	.13	− .09
4. Environmental QoL	.47***	.35***	.04	–	.08	.06	.22*	− .01
5. DS	− .19	− .29**	.00	− .22*	–	− .08	.02	.06
6. SoC	.31**	.25**	.01	.19	− .34**	–	.04	.13
7. Strength	.04	.10	.07	.16	− .10	.05	–	− .28*
8. Functional capacity^a^	.07	.08	− .01	− .00	− .09	.06	− .19	–

*p < .05, **p < .01, ***p < .001

^a^Negative change means better functional capacity

## Discussion

The purpose of this study was to determine the effects of RT on QoL, SoC, and DS, as well as to compare the possible effects of different training frequencies on those variables. After a 3-month training period, there was an intervention effect on environmental dimension of QoL. From month-3 to month-9, environmental QoL decreased among those who trained once a week compared to higher training frequencies. Throughout the 9-month intervention, participants who trained twice a week improved their environmental QoL compared to all other groups and SoC compared to CG and to RT3. Therefore, our hypothesis regarding the positive effect of RT on psychological outcomes was partly supported.

We found an intervention effect on environmental QoL after 3 months of training. This is a novel finding, partly because the environmental dimension of QoL is not part of commonly used health-related QoL measurements (e.g., RAND-36/SF-36). Bonganha et al. [22] used the same WHOQOL-BREF questionnaire, and did not observe any changes in environmental QoL after a 16-week 3-times-a-week RT program, but their participants were younger (post-menopausal women) than in the present study. The environmental QoL reflects how satisfied individuals are with their environment and with their access to different services. In the present study, the change in environmental QoL during the first 3 months correlated positively with changes in maximum strength, so it is possible that initial improvements in strength contributed to the participants’ abilities to use their environment. Environmental dimension of QoL includes questions related to home environmental, physical safety, but also (importantly in the context of the present study) the individual’s possibility to access leisure activities, health services, and public transport [1]. It is possible that this dimension measures partly similar concepts related to improved functional capacity as the physical dimension, which was borderline statistically significant after 3 months of training. While there was a positive relationship between changes in environmental QoL and strength, the relationship was weak (*r* ^2^ = 0.04), and no relationships were noted for changes in functional capacity. Consequently, the satisfaction of one’s own capabilities in relation to the environment seems not entirely dependent on actual physical changes.

Regarding SoC, the results suggest that SoC improved in the training group after 3 months of training, but the change was not statistically significant compared to CG. However, when comparing the change from baseline to month-9, SoC improved among RT2 compared to CG and RT3. According to these results, it seems that changes in SoC develop slowly, and that the RT intervention needs to be longer than 3 months. SoC is described to be stable and enduring, but not an unchangeable life orientation [6, 40, 41]. Exercise is one of the resources contributing to good SoC [6, 42, 43]: it seems that physical activity can contribute to SoC, but it has to be regular and continuous, part of a lifestyle, to bring about a change. Both SoC and environmental QoL are important health-promoting resources and closely related to overall well-being of older adults [1, 8]. As the results of the present study indicate that RT is a potential way to improve these relative stable constructs, the importance of RT in promoting both physical and psychological health should be better taken into account.

Previously, two studies observed no differences in the change in QoL between RT frequencies [28, 29]. The results of the present study showed that training twice a week was the most effective frequency to increase environmental QoL and SoC, which were not measured in those previous studies. It seems that improvements in environmental QoL and SoC could not be solely due to improvements in physical functioning and strength; otherwise, also RT3 should have improved during the 9-month period. One explanation for this could be that among previously sedentary older adults, three high-intensive RT sessions per week were too much for their psychological functioning: for example, meta-analysis by Arent et al. [44] showed that exercise interventions with training frequency less than three times a week were more beneficial to older adults’ mood than interventions with three or more session per week. Another explanation could be regularity and continuity: perhaps continuing with the same training frequency throughout the intervention was the key element for improvements in RT2. It is possible that a reduced training frequency for the last 6 months have led to the feeling of the loss of benefits, whereas increasing training frequency may have been perceived as too much. It could be speculated that continuing RT with the same frequency over 9 months may have offered a sufficient feeling of continuity. In future, studies investigating the effect of different training frequencies on different areas of psychological functioning should start the intervention directly with different frequencies to determine possible between-group differences from the initiation. In addition, it would be important to investigate the effect of changes in training frequency in relation to psychological functioning, because it is quite common in practice to have, e.g., seasonal variation in training [45].

During the initial 3 months of RT, physical and psychological QoL increased and DS decreased in the training group, and in psychological QoL and DS these changes remained to the end of the intervention. These findings are in line with some previous studies, where both shorter (≤ 3 months) [18, 19, 46–48] and longer (≥ 6 months) [20, 25, 49] RT interventions have had positive effects on physical and psychological QoL and DS. On the other hand, RT does not affect QoL according to some previous studies [21–23]. The inconsistent results regarding QoL may be due to differences between studies: for instance, in studies where no intervention effect on QoL occurred [21–23], participants where younger than in those studies where improvements were observed [18–20, 25, 46–49]. Damush and Damush [23] observed that both training and control group seemed to improve in health-related QoL measurements, possibly because the control group was also allocated to social interaction. In the present study, CG was not allocated to social interaction, but they increased their aerobic exercise despite the instructions not to change their lifestyle. These findings may indicate that the participants in the present study were all motivated to improve their health/well-being and may have influenced the findings. For instance, a decrease in DS occurred also in CG. Although the GEE analyses were adjusted by the amount of aerobic training, this lifestyle change may explain why there were no intervention effects on these variables.

These results also suggest that apart from SoC, the largest changes in QoL and DS seemed to occur in the beginning of RT. This is in line with the results of two meta-analyses showing that shorter exercise programs are more effective for psychological functioning than longer ones among older adults [14, 44]. It is possible that individuals perceive physical benefits of exercise even after short-term training and these improvements in turn contribute to better psychological functioning [14]. This would seem logical given that the largest gains in physical function occur at the beginning of a training intervention and these gains plateau after some months [12], as was also the case in this intervention [31, 32]. In the present intervention, correlations indicate that improvements in strength are slightly associated with psychological functioning after 3 months of intervention, but not after 9 months. It seems that, especially longitudinally, the relationship between exercise and psychological functioning is more complicated; in addition to improvements in physical functioning, there are many other possible mediators that could explain the relationship [17]. Social interaction, master experiences and self-efficacy, stress-removal, and hormonal changes are possible mediators [14, 17]. These mechanisms between RT and psychological functioning may be a fruitful area for future research, since they are not well understood. There is evidence that psychological improvements return to baseline after an intervention, especially among those participants who do not continue RT after the intervention [19, 50]. Therefore, it is important to encourage older adults to participate in RT after the intervention to maintain improvements in psychological functioning also.

The results of this study show that RT influences some areas of psychological functioning but not all. It is also possible that high baseline scores have produced a ceiling effect. At baseline, the QoL domain scores were higher than average in the corresponding age group [2], SoC scores were in the upper part of a range found in a systematic review [35], and the amount of DS was low in the present study with only 2% scoring over 13 (threshold for mild depression [36]). It is probable that individuals with normal levels of functioning and high baseline health-related QoL scores may not benefit from exercise as much as those with lower baseline scores [13]. In addition, this was a secondary analysis of randomized controlled trial and the power analyses were based on muscle strength and functional capacity, so it is also possible that the trial was unpowered to detect changes in psychological functioning. Nevertheless, previous studies have found significant changes in psychological functioning with similar or even smaller sample sizes [13, 28]. Despite these possible limitations, we still observed changes in environmental QoL and SoC, which gives confidence that these observations reflect true phenomena derived from the RT intervention of the present study. The high baseline scores may also indicate that the present study sample consisted largely of older adults with good psychological functioning and motivation to start training. It is not clear why those with low QoL, weak SoC, and DS did not register to participate in the RT intervention of the present study. In addition, the sample consisted of healthy older adults aged 65–75; hence, the results should be replicated among participants with a wider age range and different patient groups.

In conclusion, the key observation of this study is that, in addition to well-known physical benefits for aged populations, RT is beneficial for environmental QoL and SoC. Future studies should investigate the cause(s) of these improvements in psychological functioning; for instance, are they consequences of changes in physical characteristics or other psychological constructs? Future trials should also consider training frequency and duration in relation to RT and psychological functioning: identifying when the changes occur, and the appropriate intervention duration and quantity needed to gain the benefits.
